# Hydrolase-Treated Royal Jelly Attenuates H_2_O_2_- and Glutamate-Induced SH-SY5Y Cell Damage and Promotes Cognitive Enhancement in a Rat Model of Vascular Dementia

**DOI:** 10.1155/2021/2213814

**Published:** 2021-10-05

**Authors:** Nualpun Sirinupong, Worrapanitch Chansuwan, Pratchaya Kaewkaen

**Affiliations:** ^1^Faculty of Agro-Industry, Prince of Songkla University, Hat Yai, Songkhla 90110, Thailand; ^2^College of Research Methodology and Cognitive Science, Burapha University, Mueang, Chonburi 20131, Thailand; ^3^Animal Cognitive Neuroscience Laboratory (ACoN), Cognitive Science & Innovation Research Unit (CSIRU), Burapha University, Mueang, Chonburi 20131, Thailand

## Abstract

Vascular dementia (VaD) is the second most common type of dementia following Alzheimer's disease, but the therapeutic efficacy is still not effective. This makes the searching for novel neuroprotective agents important. Therefore, we hypothesized that royal jelly, a well-known traditional medicine, could attenuate memory impairment and brain damage in vascular dementia. This study determined the effects of royal jelly hydrolysate (RJH) and possible mechanism of cell damage and cognitive-enhancing effect in animal study. An *in vitro* study assessed the effects of RJH on acetylcholinesterase inhibitor, cell viability, and cell damage in SH-SY5Y neuroblastoma cells. Then, an *in vivo* study examined vascular dementia by the occlusion of the right middle cerebral artery (Rt.MCAO); adult male Wistar rats had been orally given RJH at doses ranging from 10, 50, to 100 mg/kg for 14 days before and 14 days after the occlusion of Rt.MCAO to mimic the VaD condition. Rats' spatial memory was evaluated using Morris water maze and radial arm maze every 7 days after Rt.MCAO throughout a 14-day experimental period, and then, they were sacrificed and the acetylcholinesterase (AChE) activity in the hippocampus was determined. The results showed that RJH has no cytotoxic effect with the final concentration up to 500 *μ*g protein/ml and reduces cell death from the H_2_O_2_- and glutamate-induced cell damage in *in vitro* neuroblastoma cells. Importantly, RJH significantly improved memory performance in Morris water maze test and radial arm maze and decreased the level of acetyl cholinesterase activity. In conclusion, RJH is the potential neuroprotective agent and cognitive enhancer for VaD.

## 1. Introduction

Many countries around the world currently are facing an aging society duet to low birth rate and longer life expectancy. According to the World Health Organization (WHO), the number of senior populations aged 60 years or older is increasing at least by 3% per year which can reach 3.1 billion in 2100. Thailand's aging population is on par with many developed countries; it is ranked as the third rapidly aging population in Asia, which is expected to be 26.9% of the total population in 2030, which is equivalent to a quarter of the overall population. Aging, a natural phenomenon of life, is the deterioration of the body over time which is therefore causing various diseases by the degeneration of various organs, especially the brain. The brain is the most complex part of the human body that controls our thoughts, memory, and speech, including intelligence, movement of the arms and legs, interpretation of the senses, and behavior, which is the source of all the qualities that define our humanity [1]. Aged brains and neuron cell injury become highly prone to neurodegenerative diseases such as Alzheimer's disease (AD) and Parkinson's disease (PD) in which there is deterioration in memory, thinking, behavior, moving, and the ability to perform everyday activities, as well as dementia, a syndrome usually of a chronic or progressive nature in which there is deterioration in cognitive function beyond that might be expected from normal aging. It affects memory, thinking, orientation, comprehension, calculation, learning capacity, language, and judgments [2].

According to the generation of free radical and oxidative stress theory of aging [3, 4], the imbalance between endogenous and exogenous reactive oxygen and nitrogen species (RONS) accumulation and their antioxidant defence processes causes oxidative stress which promotes cellular senescence and apoptosis. The oxidative stress theory of aging is based on the hypothesis that age-associated functional losses are due to the accumulation of RONS-induced damages. At the same time, oxidative stress is involved in several age-related conditions including neurodegenerative diseases [5]. Therefore, the use of free radical scavenging molecules for the reduction of intracellular reactive oxygen species is one of the strategies used in the clinical management of neurodegeneration.

One of the important factors in achieving slowed down deterioration of organs and the brain is diet. Nowadays, people are turning to pay more attention to their health and interest in consuming functional foods and dietary supplements. Royal jelly is one of the most popular and valuable natural products derived from the hypopharyngeal glands of honeybees (*Apis mellifera*). From the past, royal jelly is mostly used as traditional medicine, long-lived health food, cosmetics, and dietary supplements. Several studies reported that royal jelly exhibits various pharmacological effects including antiaging, antioxidants, neurotrophic, anti-inflammatory, immunomodulating, hypoglycemic, and general tonic. Therefore, it is commonly used as a supplement for diseases such as cancer, diabetes, and cardiovascular dementia, and royal jelly has shown significant neuroprotective actions [6]. It has been demonstrated that royal jelly has a beneficial role in neural functions and was investigated on spatial learning and memory in a rat model of streptozotocin-induced sporadic Alzheimer's disease [7]. Moreover, royal jelly long-term administration can affect the brain neurotransmitters in naturally aged rats [8]. In addition, RJ extract facilitates neurogenesis by increasing the differentiation of neural stem cells into different types of brain cells, including neurons [9]. In a study, oral treatment with royal jelly on an icv-STZ rat model of sporadic Alzheimer's disease decreased brain glucose and energy metabolism, cognitive impairment, oxidative stress, neuronal loss, and amyloid angiopathy [10].

However, although royal jelly has many benefits, there are some consumers who are allergic to royal jelly, especially those with respiratory diseases such as asthma. The substances that cause allergies in royal jelly are proteins. There are researches and application which reported that breaking down proteins into peptides such as digestion by enzyme is able to reduce the activity of allergenic sensation. Our previous study showed that the enzymatic-treated royal jelly hydrolysate attenuates IgE antigen-mediated allergic reaction [11]. Therefore, in this study, an animal model was designed to examine the effect of the hydrolase-treated royal jelly on neurons protecting *in vitro* and *in vivo*, including the effects on enhancing learning, memory, and recall of old memory regeneration of damaged neurons in normal rat and model of vascular dementia.

## 2. Materials and Methods

### 2.1. Material

Three batches of pure fresh royal jelly (RJ) from the honeybee species *Apis mellifera* were acquired directly from beekeepers in Northern Thailand. RJ was transferred by a refrigerated truck and kept frozen (−20°C). The storage period of samples used in this study was not more than six months.

### 2.2. Chemical and Reagents

Human neuroblastoma SH-SY5Y cells (ATCC® CRL-2266™) were from ATCC, USA. Minimum essential medium eagle (MEM), 3-(4,5-dimethyl-2-thiazolyl)-2,5-diphenyl-2H-tetrazolium bromide (MTT), and purified acetylcholine esterase (AChE) were purchased from Sigma. Fetal calf serum (FCS) was from Gibco. QuantiChrom™ Acetylcholinesterase Inhibitor Screening Kit (IACE-100) was from BioAssay Systems, USA. All chemicals used in the experiments were of analytical grade. Donepezil (Aricept®) is a standard drug used for mild cognitive impairment treatment.

## 3. Method

### 3.1. Preparation of Royal Jelly Hydrolysate (RJH)

Royal jelly was hydrolyzed by enzymes, according to a previous work [11]. Briefly, the optimum condition used for hydrolysis was based on the recommended conditions from the supplier. The RJ samples were mixed with deionized water at a ratio of 1 : 2. The reaction was performed in constant agitation at 200 rpm, and the protease was added at a level of 1.0% (*w*/*w*). Temperature was stabilized with a continuous flow water bath. The hydrolysis was carried out for four hours; the reaction was terminated by heating at 85°C for 15 min before cooling on ice. The hydrolysate was then centrifuged at 10,000 × *g* at 4°C for 15 min. The soluble fraction was collected and lyophilized then kept at −20°C for further analysis.

### 3.2. Determination of *In Vitro* AChE Inhibitor

Acetylcholine esterase inhibition was performed by applying the method from the Ellman method [12] and used a ready-made reagent kit, QuantiChrom™ Acetylcholinesterase Inhibitor Screening Kit (IACE-100) Rapid Colorimetric Screening for Acetylcholinesterase Inhibitors (BioAssay Systems, USA). The samples used in the experiment are RJH hydrolyzed with 3 enzymes at different hydrolysate times. The AChE enzyme activity was determined based on the reactions below. The intensity of the resulting yellow product was measured at 412 nm which was proportional to the enzyme activity presented in the sample. Donepezil (AChE inhibitor) (a medicine used for Alzheimer's disease treatment) was used as a positive control. Typically, a neurotransmitter such as acetylcholine (Ach) plays a role in transmission of nerve impulse within the brain essential for a person's memory. If the amount of acetylcholine is reduced, it can lead to dementia.

(1) $$\begin{array}{c} \text{Acetylthiocholine}\overset{\text{ACh}}{⟶}\text{Thiocholin}+\text{Acetate Thiocholin}+\text{Dithiobisnitrobenzoate}⟶\text{Yellow} \end{array}$$

### 3.3. Cell Culture

The human neuroblastoma, SH-SY5Y, cell suspension was cultured in 37°C with EMEM and F12 medium in the incubator under 5% CO_2_ conditions. The medium contained 3.7 mg/ml sodium bicarbonate, 10% fetal bovine serum, and antibiotics penicillin G sodium (100 units/ml) and streptomycin (100 *μ*g/ml) for 5–7 days. After that, cells were trypsinized by 0.25% trypsin-EDTA. The cell suspension was centrifuged at 1200 rpm at 20°C for 5 minutes, and then, the cell pellet was gently dissolved in DMEM containing 10% FBS. The number of living cells was counted by trypan blue exclusion method. 10 *μ*l of cell suspension was suspended with 10 *μ*l of trypan blue dye and 10 *μ*l was transferred into a haemocytometer, and the cells were seen under a microscope. The cells were then cultured for next passage in a new 75 cm^2^ cell culture flask to increase the number of cells. The medium was replaced every 4–5 days, and the cells were harvested when there is approximately 80–90% on the flask area.

### 3.4. Cell Viability by MTT Assay

A SH-SY5Y cell viability assay was performed using the MTT colorimetric method according to previously described study [11]. Briefly, after 48 h incubation with the test samples, MTT solution (10 *μ*l, 5 mg/ml in PBS) was added to the wells. After 4 h incubation, the medium was removed and DMSO was then added to dissolve the formazan production in the cells. The optical density of the formazan solution was measured with a microplate reader at 570 nm. The test samples were considered cytotoxic when the optical density of the sample-treated group was less than 80% of that in the control (vehicle-treated) group.

### 3.5. Determination of Neuroprotective Effect on H_2_O_2_- and Glutamate-Induced Cell Damage

#### 3.5.1. Determination of the Appropriate Concentration of H_2_O_2_ and Glutamate

The neuroprotective effect of RJH was determined on human neuroblastoma SH-SY5Y cell damage induced by H_2_O_2_ and glutamate. 30% H_2_O_2_ solution (9.794 M equivalent) was diluted in phosphate-buffered saline (PBS; Sigma-Aldrich, USA) to obtain various concentrations of 25, 50, 100, 200, 300, 400, and 500 *μ*M. Then, each concentration was added into the 48 h cultured SH-SY5Y cell at amounts of 3 × 10^4^ cells/cm^2^. The treated cell was continuously cultured for another 24 h. The control-treated cell was added PBS instead of H_2_O_2_. Then, the cell viability was determined using MTT assay as previously described. The concentration of H_2_O_2_ that allows 50% cell survival was selected for further inducing cell damage. The optimum concentration of glutamate that led to 50% SH-SY5Y cell viability would be used for inducing cell damage, and a similar method was performed for H_2_O_2_. The glutamate solution was diluted with PBS to obtain the various concentrations of 1, 10, 25, 50, 75, and 100 mM and then used for cell induction.

#### 3.5.2. Determination of Neuroprotective Effect

The neuroprotective effect of RJH against H_2_O_2_- and glutamate-induced cell damage was tested with the cultured SH-SY5Y at an amount of 3 × 10^4^ cells/cm^2^, for 48 hours. The treatment was divided into two groups, cotreatment and pretreatment. In cotreatment, RJH samples were added with the appropriated concentration of H_2_O_2_ or glutamate solution into the cell while in pretreatment RJH samples were added into the cell and incubated at 37°C under 5% CO_2_ condition for 6 hours before adding H_2_O_2_ or glutamate solution; then, the treated cells were continually cultured for another 24 hours. The cell viability was then assessed by MTT assay. The final concentration of H_2_O_2_ and glutamate of 200 mM and 50 mM, respectively, was used to induce cell damage. The final concentration of Flavourzyme-treated RJH used to treat cell was 200 *μ*g protein/ml.

### 3.6. Determination of Behavioral Study in Animal's Study

#### 3.6.1. Animals

Adult male Wistar rats (250–300 g, 8 weeks old) were obtained from Nomura Siam International Co., Ltd. and were housed in groups of 5 per cage in standard metal cages at 22 ± 2°C on a 12 : 12 h light-dark cycle at the Cognitive Neuroscience Laboratory. All animals were given access to food and water *ad libitum*. The experiments were performed to minimize animal suffering in accordance with the internationally accepted principles for laboratory use and care by the Institutional Animal Care and Use Committee, Burapha University, Thailand (26/2562).

#### 3.6.2. Experimental Protocol Design

In the part of animal's study, we followed the methods of Kaewkaen et al. [13]. All rats were randomly divided into 5 groups. Each group contained 8 rats.

Group 1: vehicle-treated group: the animals in this group were treated with propylene glycol (PG)

Group 2: positive control-treated group: the positive control group was treated with the standard drugs used for treating the related condition. The positive control group was treated with donepezil (Aricept®, a cholinesterase inhibitor) at a dose of 1 mg/kg

Groups 3–5: treated group: the animals in groups 3–5 were treated with the RJH at various doses ranging from 10, 50, to 100 mg/kg, respectively, via oral route administration for two weeks once daily throughout the experimental period and two weeks after inducing middle cerebral artery occlusion

### 3.7. *In Vivo* Evaluation of Vascular Dementia

In this part, rats underwent a surgical procedure to induce middle cerebral artery occlusion (MCAO) to mimic vascular dementia. The focal cerebral ischemia induction was performed according to the modified method of Longa et al. [14]. In brief, rats were anesthetized with thiopental sodium at a dose of 50 mg/kg BW. The right common carotid artery and the right external carotid artery were exposed through a ventral midline neck incision and were ligated proximally. A silicone-coated nylon monofilament (4-0) suture (USS DGTM sutures; Tyco Healthcare Group LP, Connecticut, USA) with its tip rounded by heating near a flame was inserted through an arteriectomy in the common carotid artery just below the carotid bifurcation and then advanced into the internal carotid artery approximately 17–18 mm distal to the carotid bifurcation until a mild resistance was felt. Occlusion of the origins of the anterior cerebral artery, the middle cerebral artery, and the posterior communicating artery was thereby achieved. Then, the wound was sutured; the rats were returned to their cages with free access to food and water. The incision sites were infiltrated with 10% povidone-iodine solution antiseptic postoperative care.

#### 3.7.1. Assessment of Cognitive Function by Morris Water Maze (MWM)

The animals' spatial memory was tested by the water maze test [15]. The apparatus was a pool with 170 cm diameter filled up with tap water for 40 cm deep, and the water surface was covered with nontoxic powder. The pool was divided into four quadrants, and the removable escape platform was placed in the centre on one quadrant below the water level. For animals, the location of the platform was invisible and it remained there throughout the training. The animals must memorize the environment cues to locate the platform. Each animal was placed in the water in the starting quadrant and allowed to swim until it found and climbed onto the platform. Each rat was gently placed in the water facing the wall of the pool from one of the four starting points along the perimeter of the pool, and the animal was allowed to swim until it found and climbed onto the platform. During the training session, the rat was gently placed on the platform when it could not reach the platform in 60 seconds. In either case, the subject was left on the platform for 15 seconds and then removed from the pool. The time for the animal to reach the hidden platform was recorded as escape latency or acquisition time. Approximately twenty-four hours later, each animal was subjected to the identical conditions as before with the exception of the removal of the submerged platform. This study also assessed the amount of time that each animal spent swimming in the quadrant that had previously housed the submerged platform also known as retention time.

#### 3.7.2. Assessment of Cognitive Function by Radial Arm Maze (RAM)

The radial arm maze is an apparatus consisting of a circular centre compartment from which 8 equally spaced (arms) extend. Each arm is an elongated column of equal distance. The short-term working reference memory was examined in rats by using a radial arm maze with 8 arms (9 cm × 63 cm) originating from a central platform (30 cm wide octagon). A reward cup containing a single sucrose pellet was located at the distal end of each arm. Each rat was individually placed on the RAM daily for 7 days and allowed to explore the maze until all eight arms had been visited or until 6 min expired. At the completion of this acquisition training period, all animals completed the maze with less than 1 working error as indicated by a return to a previously visited arm on the same trial [16].

### 3.8. *In Vivo* Acetylcholinesterase Assay

The RJH-treated rats were sacrificed, and then, the brains were isolated and kept cool in ice buckets. Then, the tissues of the hippocampus were homogenized in 4 volumes of 1.15% KCl with glass Potter-Elvehjem homogenizer. An AChE assay was performed using the colorimetric method [12] with minor modifications. The hippocampus was homogenized in 0.1 M phosphate buffer, pH 8.0. The reaction mixture consists of 2.6 ml of 0.1 M phosphate buffer pH 8.0, 0.4 ml aliquot of homogenate, and 0.1 ml of 0.01 M dithiobisnitrobenzoic acid (DTNB). The substrate 0.075 M acetylthiocholine iodide was added, and the change in the absorbance was noted every 2 min for 10 min at 412 nm using a spectrophotometer. The AChE activity was expressed as micromoles hydrolyzed per milligram protein of tissue (*μ*mol/mg protein).

### 3.9. Statistical Analysis

Data were subjected to analysis of variance (ANOVA) and Duncan's multiple range test. The SPSS statistics program (version 16.0) was used for data analysis. Statistical significance was tested at *p* < 0.05.

## 4. Results and Discussion

### 4.1. *In Vitro* Acetylcholine Esterase Inhibition of RJH

To screen the optimal digestion condition for neuroprotective activity of RJH, the Acetylcholinesterase (AChE) Inhibitor Screening Kit (IACE-100) Rapid Colorimetric Screening modified method of Ellman method (1961) was performed. Donepezil (AChE inhibitor) was used as a positive control [17]. AChE is responsible for accelerating the breakdown of acetylcholine at the synaptic space inside the brain. Acetylcholine is an essential neurotransmitter for human memory and cognitive function if the amount of this neurotransmitter is diminished resulting in dementia such as Alzheimer's disease [18].

**Figure 1:**
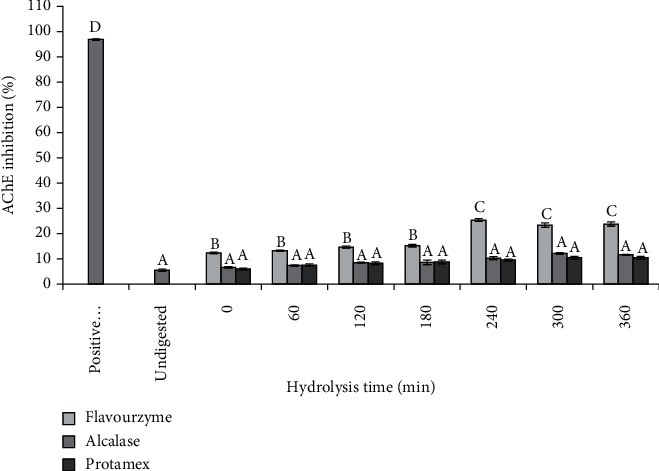
Acetylcholinesterase inhibitory capacity of three enzyme-treated royal jelly hydrolysates. The different letters have mean values that are significantly different (*p* < 0.05) compared to the positive control and unhydrolyzed groups.

The optimal digestion condition including enzyme type and hydrolysate time on AChE inhibitory activity is showed in Figure 1. The result showed that RJH treated with Flavourzyme has ability to inhibit AChE significantly higher than RJH treated with Alcalase and Protamax at the hydrolysate time of 240–360 minutes. Flavourzyme-treated RJH exhibited an AChE inhibition percentage of 24–26%, which was the most effective inhibition. However, the ability of peptides presented in RJH against AChE inhibition was less than that of the positive control, donepezil, which showed about 98% AChE inhibition. There is an abundance of natural peptides from different sources which have positive impacts on neurodegenerative preventions and treatments through AChE inhibitory activity including terrestrial venomous animal peptides, plant peptides, marine peptides, and venomous amphibian peptides [19]. A peptide from Ziziphus jujuba fruits was investigated for its inhibitory activity against AChE and butyrylcholinesterase (BChE) enzymes as well as antioxidant activity [20]. Recently, hemp seed peptides targeting AChE inhibitory enzymatic hydrolysates were obtained using six distinct proteases. The pepsin hemp seed peptides showed higher AChE inhibitory effects [21]. After that, the acetylcholinesterase (AChE) inhibitory activity of pepsin-produced hemp seed protein hydrolysates (HPH) was enhanced through reverse-phase HPLC separation followed by identification of peptide sequences present in the most active fraction. The HPH consisted mostly of low molecular weight peptides of <11 amino acid residues and mostly contained at least one hydrophobic amino acid [22]. There is identification of the polysaccharide-peptide complex in AChE inhibitory activities from the mushroom *Cordyceps militaris* (CPSP) and characterization of AChE inhibitory properties [23]. Anchovy protein hydrolysates (APHs) showed antioxidant activity and AChE inhibitory activity, and in vivo mouse tests showed that all APHs exhibited memory-improving action on scopolamine-induced amnesia mice [24]. The AChE inhibitory properties of Hp-1935, a natural peptide extracted from the skin secretions of an Argentinian frog (*Boana pulchella*), were studied [25]. Moreover, synthetic peptides were reported to exhibit anti-AChE activity such as an octapeptide which interacts with the catalytic anionic site (CAS) of AChE enzyme and inhibits AChE activity [26]. Another study showed five peptide amide and ester analogues of galanthamine, AD's medicine [27].

### 4.2. Neuroprotective Effect of RJH against H_2_O_2_- and Glutamate-Induced Cell Damage

**Figure 2:**
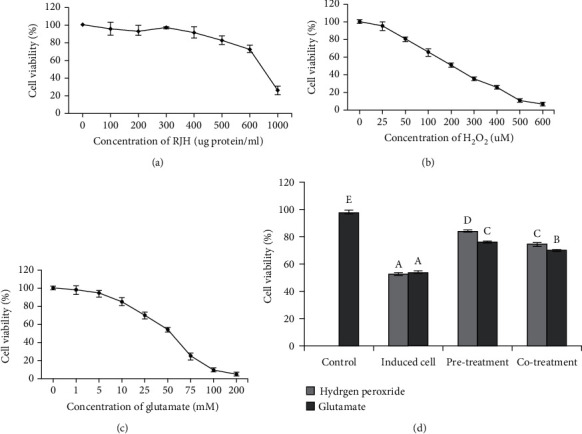
Cytoprotective neuroprotective activity of Flavourzyme-treated RJH at 240 min hydrolysate by pretreatment and cotreatment of hydrogen peroxide- and glutamate-induced SH-SY5Y cell damage. The effect of (a) RJH, (b) H_2_O_2_, and (c) glutamate on SH-SY5Y cell viability and (d) the protection of RJH on H_2_O_2_- and glutamate-induced cell damage after 24 hours of incubation. The different letters have mean values that are significantly different (*p* < 0.05).

SH-SY5Y cells were employed to evaluate the cytoprotective effect of Flavourzyme-treated RJH against oxidative stress-induced damage. First, SH-SY5Y cells were treated with various final concentrations of RJH at 0–500 *μ*g protein/ml to evaluate their potential cytotoxic effect by MTT assay. The result showed that RJH has no cytotoxic effect at the final concentration up to 500 *μ*g protein/ml (Figure 2(a)). The SH-SY5Y cell viability by MTT assay also was determined for the appropriate concentration of H_2_O_2_ and glutamate at different concentrations as shown in Figures 2(b) and 2(c); as the result displays, as the concentration of H_2_O_2_ and glutamate increased, cell vitality decreases and continues to drop to less than 50% when the concentration was more than 200 *μ*M for H_2_O_2_ and 50 mM for glutamate. In order to see the effects that RJH peptides could protect cell damage subsequently, the final concentrations of 200 *μ*M H_2_O_2_ and 50 mM glutamate were selected to induce oxidative damage of the cells with the RJH final concentration of 200 *μ*g protein/ml.  Figure 2(d) shows that SH-SY5Y cells pretreated with RJH before being exposed to oxidative stress by H_2_O_2_ or glutamate significantly survived than SH-SY5Y cells cotreated with RJH and exposed to oxidative stress by H_2_O_2_ or glutamate. The result suggested that RJH peptides could recover the reduction of cell viability from H_2_O_2_- and glutamate-induced cell damage.

### 4.3. Cognitive-Enhancing Effect of RJH by MWM

**Figure 3:**
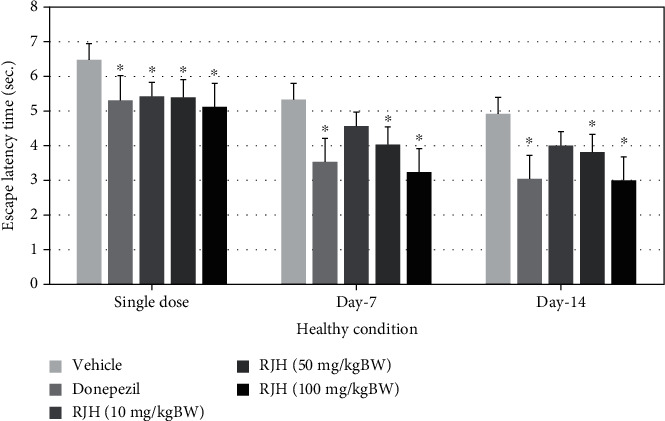
The effect of RJH on escape latency time in healthy condition. Rats were treated with vehicle, donepezil, or RJH in various doses ranging from 10, 50, to 100 mg/kg BW via oral route for 14 days; then, their escape latency time was determined in the Morris water maze test. The test was performed within 30 minutes after the substance administration. Data are presented as mean ± SEM (*n* = 8/group). ^∗^*p* < 0.05 compared with the vehicle-treated group.

**Figure 4:**
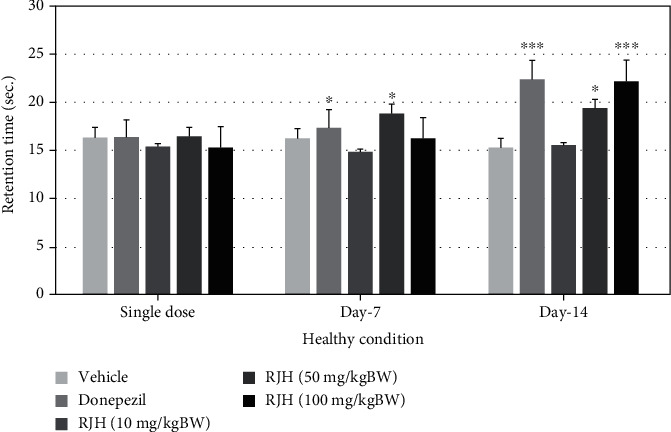
The effect of RJH on retention time in healthy condition. Rats were treated with vehicle, donepezil, or RJH in various doses ranging from 10, 50, to 100 mg/kg BW. Data are presented as mean ± SEM (*n* = 8/group). ^∗^*p* < 0.05 compared with the vehicle-treated group; ^∗^*p* < 0.05 and ^∗∗∗^*p* < 0.001 compared with the vehicle-treated group.

The effect of RJH on spatial memory, hippocampal-dependent memory, was determined using the Morris water maze test [15]. Rats were treated with vehicle or donepezil (1 mg/kg) or RJH at various doses ranging from 10, 50, to 100 mg/kg via oral administration route. The rats' escape latency was determined in the Morris water maze test within 30 minutes after substance administration, and 24 h later, they were also exposed to the same test and the retention time is determined and shown in Figures 3 and 4.

The present results demonstrated that donepezil-treated rats have significantly decreased escape latency both after single administration and at 7 days of treatment (*p* < 0.05, compared with the control group). Interestingly, rats treated with RJH at all doses also showed significant reduction of escape latency both after single administration (*p* < 0.05 all, compared with the control group) and at 14 days of treatment shown 50 and 100 mg/kg (*p* < 0.05, compared with the control group).

**Figure 5:**
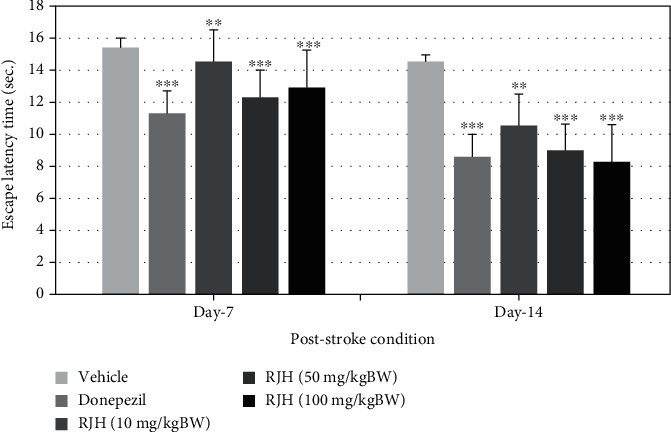
The effect of RJH on escape latency time in stroke condition. Rats were treated with vehicle, donepezil, or RJH in various doses ranging from 10, 50, to 100 mg/kg BW via oral route for 14 days; then, their escape latency time was determined in the Morris water maze test. The test was performed within 30 minutes after the substance administration. Data are presented as mean + SEM (*n* = 8/group). ^∗^*p* < 0.05 compared with the vehicle-treated group.

**Figure 6:**
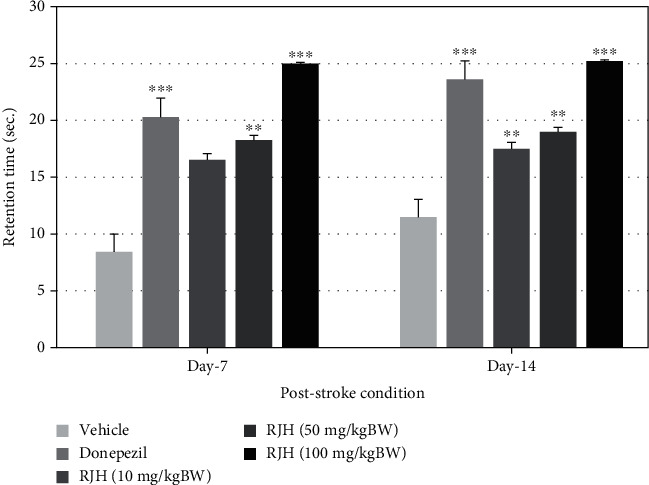
The effect of RJH on retention time in stroke condition. Rats were treated with vehicle, donepezil, or RJH in various doses ranging from 10, 50, to 100 mg/kg BW. Data are presented as mean ± SEM (*n* = 8/group). ^∗^*p* < 0.05 compared with the vehicle-treated group; ^∗∗^*p* < 0.01 and ^∗∗∗^*p* < 0.001 compared with the vehicle-treated group.

The effect of RJH on spatial memory in VD condition by MCAO was determined using the Morris water maze test. Rats were treated with vehicle or donepezil (1 mg/kg) or RJH at various doses ranging from 10, 50, to 100 mg/kg via oral administration route. The rats' escape latency was determined in the Morris water maze test within 30 minutes after substance administration, and 24 h later, they were also exposed to the same test and the retention time is determined and shown in Figures 5 and 6.

The results demonstrated that donepezil-treated rats have significantly decreased escape latency both after administration at 7 days of treatment after MCAO (^∗∗^*p* < 0.01 and ^∗∗∗^*p* < 0.001 compared with the vehicle-treated group). Rats treated with RJH at all doses also showed significant reduction of escape latency in 14 days after MCAO (^∗∗^*p* < 0.01 and ^∗∗∗^*p* < 0.001 compared with the vehicle-treated group).

### 4.4. Cognitive-Enhancing Effect of RJH by RAM

**Figure 7:**
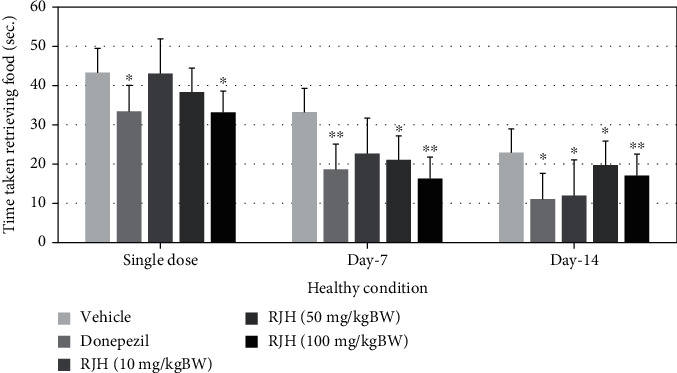
The effect of RJH on time taken retrieving food in healthy condition. Rats were treated with vehicle, donepezil, or RJH in various doses ranging from 10, 50, to 100 mg/kg BW. Data are presented as mean + SEM (*n* = 8/group). ^∗^*p* < 0.05 and ^∗∗^*p* < 0.01 compared with the vehicle-treated group.

**Figure 8:**
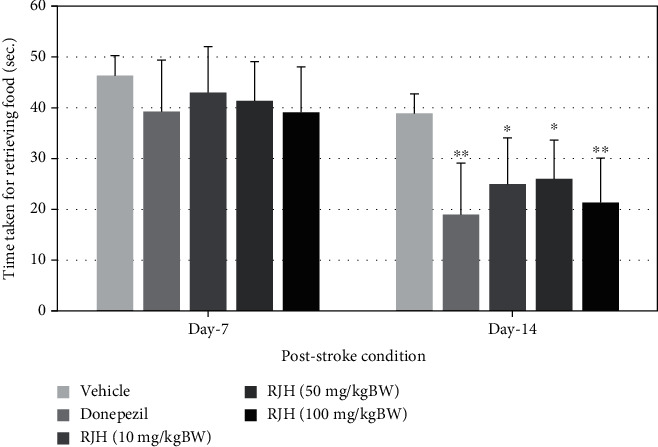
The effect of RJH on time taken retrieving food in stroke condition. Rats were treated with vehicle, donepezil, or RJH in various doses ranging from 10, 50, to 100 mg/kg BW. Data are presented as mean + SEM (*n* = 8/group). ^∗^*p* < 0.05 and ^∗∗^*p* < 0.01 compared with the vehicle-treated group.

To investigate whether the effect of RJH (10, 50, and 100 mg/kg) affects spatial memory formation, the rats were further evaluated in the radial arm maze task, indicating that the RJH significantly improved working memory during single doses, 7 and 14 days of administration (*p* < 0.05) compared with the vehicle-treated group and shown in Figures 7 and 8.

### 4.5. Effect of RJH on AChE Activity in the Hippocampus

**Figure 9:**
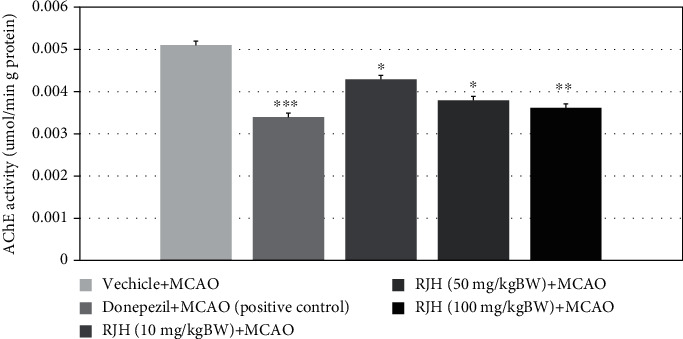
The effect of RJH on the alteration of acetylcholinesterase enzyme activity in the hippocampus. Rats were treated with vehicle, donepezil, or RJH in various doses ranging from 10, 50, to 100 mg/kg BW. Data are presented as mean + SEM (*n* = 8/group). ^∗^*p* < 0.05 and ^∗∗^*p* < 0.01 compared with the vehicle-treated group.

Rats were treated with vehicle or donepezil or RJH at doses of 10, 50, and 100 mg/kg via oral administration for a week, and Rt.MCAO was performed. Then, rats were sacrificed to determine the activity of AChE activity in the hippocampus. Data presented that all doses of RJH decrease AChE activity in the hippocampus as shown in Figure 9.

## 5. Discussion

The hydrogen peroxide and excessive glutamate (excitatory neurotransmitter) known as neurotoxin cause oxidative stress by production of excessive reactive oxygen species (ROS) [28–30]. The ROS is thought to be an important cause of cell injury resulting in various neurodegenerative diseases, such as Alzheimer's disease and Parkinson's disease [31, 32]. Cells may be protected from oxidative injury by antioxidant systems. In several studies, it has been reported that active compounds from natural products act as antioxidant on neuron cell. Neuroprotective effects of hydrolysates and peptides have been demonstrated. As similar result shown in this study, RJH reduced cell death from the H_2_O_2_- and glutamate-induced cell damage. A study by Zhang et al. [33] reported that the neuroprotective effect of hydrolysate from whey protein increases viability of PC12 cells under hydrogen peroxide-induced damage. The two antioxidant peptides were identified and characterized from the alcalase hydrolysate of housefly (*Musca domestica L*.) pupae. The active peptides can effectively protect PC12 cells from oxidative damage induced by hydrogen peroxide by decreasing intracellular reactive oxygen species and malonaldehyde recovering cellular mitochondrial membrane potential and increasing the activity of intracellular superoxide dismutase guided by ABTS cation radical scavenging activity [34]. An antioxidant peptide derived from walnut meal protein and passed through gastrointestinal digestion displayed ABTS radical scavenging activity and ORAC assay also exhibited significantly protective effect on SH-SY5Y cells against oxidative damage [35]. The less than 3 kDa and 10 kDa peptide fractions obtained by commercial hydrolysis enzymes have superior neuroprotective activity and antioxidative activities in hydrogen peroxide-treated SH-SY5Y cells and did not affect cytotoxicity. In addition, the <3 kDa peptide fractions reduced DNA fragmentation and reactive oxygen species generation and increased superoxide dismutase activity and catalase levels in H_2_O_2_-treated SH-SY5Y cells. Both peptide fractions showed strong antioxidant activities in 2,2′-azino-bis(3-ethylbenzothiazoline-6-sulfonic acid) diammonium salt, iron chelation, and reducing power tests [36]. Recently, an *in vivo* research reported that the 40 oligopeptides (19 dipeptides, 1 tripeptide, 12 tetrapeptides, 6 pentapeptides, 1 hexapeptide, and 1 heptapeptide) identified from sea cucumber exhibited the increasing of spatial memory and learning; in vitro effects include inhibition of MDA and protein oxidation such as carbonyls and sulfhydryls and antioxidant enzymes (SOD and GSH-Px) [37].

The Morris water maze is one of the most widely used tasks in behavioral assessment of rodents for studying the neural mechanisms of spatial learning and memory. In this study, it was demonstrated that RJH could significantly decrease escape latency time and increase retention time in the Morris water maze test, and moreover, the level of acetylcholinesterase activity was decreased when compared with vehicle-treated groups. The result consistent with previous study showed that long-term oral administration of RJH induced beneficial effects in animals injured by icv-STZ injection, increasing retention time for working spatial memory [10]. The effect of RJH on time taken retrieving food in healthy condition when assessed in radial arm mazes shows RJH could prevent induced deficit memory in rats, as the animals showed better performance in Rt.MCAO for induced VaD and decreased AChE activity in the hippocampus tissue of the brain. A recent study reported the effect of RJ and/or its components on lifespan/healthspan in various species by evaluating the most relevant studies. Moreover, the positive effects of RJ on health maintenance and age-related disorders are evaluated [38].

The effect of occlusion in the middle cerebral artery involved with cognitive decline and progress to dementia has been reported [39]. The severity of dementia may depend on the location and magnitude of cerebral artery injury. The evidences support and describe the two most common forms of senile dementia which are Alzheimer's disease and vascular dementia. In vascular dementia relating to different vascular mechanisms which include ischemic stroke or brain hemorrhage, the clinical manifestations include memory loss, confusion and executive dysfunction, and impairment of motor control that may result from stroke [40]. The pathogenic pathways of vascular dementia are still elucidated, but many studies describe that the cholinergic pathways have the effect to decrease acetylcholine production and affect the cognitive deficits after stroke. Many studies found that the impairment of the cholinergic system involves the cortical and hippocampal area [41]. The rich content of bioactive compounds of RJH diverse health exerts benefits in this biological pathway.

## 6. Conclusions

In conclusion, an *in vitro* analysis showed that RJH obtained by enzymatic-treated royal jelly resulting in the active hypoallergenic peptides shows AChE inhibitory activity. In cultured cell experiment, RJH exerts neuroprotective effect by increasing cell viability against the oxidative stress-induced neuroblastoma cell injury. In the *in vivo* rat model, RJH significantly improved memory performance in the Morris water maze test and radial arm maze. RJH also decreases the level of acetyl cholinesterase activity in the hippocampus. Taken together, the RJH is the potential neuroprotective agent and cognitive enhancer for VaD.

## Data Availability

The data that support the findings of this study are available on request from the corresponding author.
